# Elevated CO_2_ and Water Stress in Combination in Plants: Brothers in Arms or Partners in Crime?

**DOI:** 10.3390/biology11091330

**Published:** 2022-09-08

**Authors:** Arun Kumar Shanker, Deepika Gunnapaneni, Divya Bhanu, Maddi Vanaja, Narayana Jyothi Lakshmi, Sushil Kumar Yadav, Mathyam Prabhakar, Vinod Kumar Singh

**Affiliations:** ICAR-Central Research Institute for Dryland Agriculture, Saidabad P.O., Santoshnagar, Hyderabad 500059, India

**Keywords:** elevated CO_2_, drought, photosynthesis, transpiration rate, stomatal conductance, C_4_ enzymes, malate, water deficit stress, abscisic acid, water use efficiency

## Abstract

**Simple Summary:**

The changing climate scenario envisages elevated CO_2_ (eCO_2_) and drought in many parts of the world. Elevated CO_2_ is known to increase yields in C_3_ crops like rice and wheat, on the other hand, it does not cause a similar increase in C_4_ crops like maize and sorghum. Drought is known to reduce crop growth and yield. In this mini-review we discuss the combined effects of both eCO_2_ and drought which is typical of a climate change scenario. We try and explain how C_3_ and C_4_ crops are differentially affected by these two manifestations of climate change. We specifically show how eCO_2_ in addition to its known beneficial effects can also be effective in ameliorating the effects of drought in crops. We have critically analysed the current literature and have come up with some mechanistic explanations in terms of water relations, hormonal regulation, photosynthesis and growth, nutrient uptake, and enzyme dynamics. We present here how these processes operate across a range, from ecosystem to organismal level and from molecular to the whole plant level. The information presented will help researchers to devise strategies for adaptation in crops in agricultural systems.

**Abstract:**

The changing dynamics in the climate are the primary and important determinants of agriculture productivity. The effects of this changing climate on overall productivity in agriculture can be understood when we study the effects of individual components contributing to the changing climate on plants and crops. Elevated CO_2_ (eCO_2_) and drought due to high variability in rainfall is one of the important manifestations of the changing climate. There is a considerable amount of literature that addresses climate effects on plant systems from molecules to ecosystems. Of particular interest is the effect of increased CO_2_ on plants in relation to drought and water stress. As it is known that one of the consistent effects of increased CO_2_ in the atmosphere is increased photosynthesis, especially in C_3_ plants, it will be interesting to know the effect of drought in relation to elevated CO_2_. The potential of elevated CO_2_ ameliorating the effects of water deficit stress is evident from literature, which suggests that these two agents are brothers in arms protecting the plant from stress rather than partners in crime, specifically for water deficit when in isolation. The possible mechanisms by which this occurs will be discussed in this minireview. Interpreting the effects of short-term and long-term exposure of plants to elevated CO_2_ in the context of ameliorating the negative impacts of drought will show us the possible ways by which there can be effective adaption to crops in the changing climate scenario.

## 1. Introduction

Agriculture is one of the dominant drivers of change in the Anthropocene era; at present about 11 per cent, which is about 1.5 billion ha of the total land surface area, is used for the production of crops on about 36 per cent of the land that is suitable for agriculture [1]. Agriculture is affected by the changing climate, and paradoxically, is also contributing to it. The changing dynamics in climate are the primary and important determinants of agriculture productivity. The effects of this changing climate on overall productivity in agriculture can be understood when we study the effects of individual components contributing to the changing climate on plants and crops.

There is a continuing need to feed the growing population, and globally, the human population of 7.2 billion in mid-2013 is expected to increase to almost 8.1 billion in 2025, and to further grow to 9.6 billion by 2050 [2]. Food security in terms of food availability is imperative in such a scenario. Changing climate is a reality, and slowly we are learning to adapt to it; we are also in the process of devising mitigation strategies so that we can put the brakes on the changes that in general are harmful. The competition for natural resources like land, water and energy will keep growing at a pace with which it would be difficult for us to manage unless we have sound strategies in place to adapt to the harmful effects of changing climate and further mitigate the effects of changing climate.

The effects of climate change on particular areas, specifically agriculture, are difficult to predict with a great degree of accuracy, although the overall effects are known and understood. Reports indicate that global average temperatures have increased by about 1 °C since the pre-industrial era, and that anthropogenic warming is adding around 0.2 °C to global average temperatures every decade [3]. The global CO_2_ in the atmosphere reached 407 ppm in 2018 [4]. Given the current rate of generation of CO_2_, it can be expected that it will exceed 600 ppm by the end of this century [5]. The levels of greenhouse gas (GHG) are changing rapidly; CO_2_ concentration in the atmosphere can directly affect the growth and development of vegetation in general, and it is indirectly affecting plant growth due to seasonality and variability in rainfall it causes. 

Elevated CO_2_ and drought due to low variability in rainfall are important manifestations of the changing climate. There is a considerable amount of literature that addresses these aspects in terms of effects on plant systems from molecules to ecosystems. Of particular interest is the effect of increased CO_2_ on plants in relation to drought and water stress. Increases in the source of carbon can have favourable effects on plants in relation to their growth and development, and this can be more pronounced in the presence of optimum-to-high levels of nutrients in the soil and increased water availability. These effects may be of short duration and can vary according to the photosynthetic metabolism of the plants like in C_3_, C_4_, CAM and C_3_-C_4_ intermediate plants. In addition, there are studies which show that C_3_ crops show increased growth and yield under eCO_2_ when grown under both wet and dry growing conditions. C_4_ crops show increased growth and yield only under dry growing conditions and drought leads to stomatal limitations of C_3_ and C_4_ crops and is alleviated by eCO_2_. 

The forecasts for the coming decades have projected varying changes in precipitation that can result from the increasing frequency of droughts and floods [6]. Drought is one of the important abiotic stresses in the present changing climate scenario and the study of the mechanism by which it affects plant metabolism, growth and development is of paramount importance. In the past decade, global losses in crop production due to drought totalled USD 30 billion [7]. The loss in crop production due to drought in the past ten years has been close to about 30 billion and it is estimated that about 5 billion people will be in water-scarce regions of the world by 2050, emphasising the importance of studying all the facets of drought and plant growth. Interestingly there are studies where crops are grown under field conditions, and the positive impact of elevated atmospheric CO_2_ concentrations on productivity was found to be significantly stronger under soil water limitation than under potential growth conditions, as reported in [8] for cotton, [9] for wheat, [10] for alfalfa, and also for temperate pasture species [11]. There are also evident interactive effects of elevated CO_2_ and other environmental conditions that are indicative of changing climate like drought, heat, and other stresses that invariably accompany elevated CO_2_ conditions in the atmosphere. The importance of understanding this complex relationship is imperative in a high CO_2_ atmosphere that is envisaged in future, to counter the effects of changing climate. As it is known that one of the consistent effects of increased CO_2_ in the atmosphere is increased photosynthesis, especially in C_3_ plants, it will be interesting to know the effect of drought in relation to elevated CO_2_. The possible mechanisms by which this occurs will be discussed in this minireview. Interpreting the effects of short-term and long-term exposure of plants to elevated CO_2_ in the context of ameliorating the negative impacts of drought will show us the possible ways by which there can be effective adaption for crops in the changing climate scenario.

## 2. Water Relations, Transpiration and Stomatal Conductance

### 2.1. Stomatal Dynamics

Elevated CO_2_ concentration is known to mitigate the effects of drought stress, and in a study on *Populus* spp. and *Salix* spp. by [12] it was found that when these two species were grown in ambient (350 µmol mol^−1^) or elevated (700 µmol mol^−1^) predawn water potential reduced as water stress increased, as against midday water potential which did not show any changes. The changes observed were 0.1 MPa at predawn and 0.2 MPa at midday. Increased elasticity of the cell wall is usually observed when there are altered water relations. These cellular changes allow the tress to maintain higher turgor at lower water potentials and tissue water content. The mitigating effect of higher CO_2_ was by increasing ψ_p_ at the same levels ψ_w_ which can result in osmotic adjustment. This mechanism of osmotic adjustment can improve plant metabolism or at least maintain plant metabolism at optimal levels resulting in acclimation to drought. Stomatal dynamics drive the carbon uptake during water deficit stress and when there is accompanying stress like short-term elevated CO_2_, the role of stomatal limitation in the assimilation of carbon may reduce with a reduction in photorespiration and increase in the partitioning of soluble sugars and increase in water use efficiency.

The eCO_2_-mediated regulation of stomatal conduction and transpiration rate is mainly by regulating stomatal aperture as a short-duration response [13,14,15] and other long-duration morphological modifications like changes in stomatal density [16,17]. Varied crop-specific responses were also seen [17] in stomal density where eCO_2_ increased the density of stomata in maize whereas the same decreased in Amarnath. It is interesting to note here the differences in dicot and monocot response of both the C_4_ crops. The general response observed in both the C_4_ crops is because under water deficit conditions C_4_ crops are better performing under elevated CO_2_ as they have a CO_2_ concentrating mechanism; this mechanism favours optimum photosynthesis even under lower stomatal conductance, and they can close their stomata and still perform the dark reaction with an optimum amount of CO_2_. On the other hand, the differences between dicot and monocot C_4_ plants under elevated CO_2_ may be due to the higher degree of suberization in the kranz anatomy specifically in the NADP–ME subtypes which are not seen in the dicots specifically in the NAD–ME subtype [17].

Studies on stomatal density have been indecisive in their outcomes as to what exactly is governing the decrease and increase in the density under stress conditions, although a large body of evidence says that it is one of the key morphological traits that regulates transpirational flux resistance in the leaf and conductance of stomata under eCO_2_. The underlying mechanism is shifting the balance in favour of CO_2_ uptake by increasing it under water loss conditions. On the other hand, a recent study has also suggested that stomatal density may be equally or more affected by temperature, specifically the large continental-scale geographical variations with an interplay of precipitation [18].

The mechanism of guard cell sensing of CO_2,_ especially in enriched conditions and this sensing playing a role in the turgor dynamics of the cells, has gained much acceptance in recent times; the support for this comes from the fact the CO_2_ itself is lipophilic and can easily diffuse across membranes and also move through mass flow across aquaporins. The mechanism is explained by the triggering of CO_2_ of the efflux channels of K^+^ out which in turn increases the water potential inside the cell, and this results in water moving out and in effect resulting in stomatal closure [19,20]. 

### 2.2. The ABA Conundrum

Abscisic acid (ABA) is mainly involved in the regulation of many important physiological processes in the plant at the cellular level. ABA synthesis activates many types of countering mechanisms in plants under stress, among which the main mechanism is the stomatal movement. Opening and closing are regulated in such a way that there is minimum loss of water during water deficit conditions [21,22]. The interplay of ABA and eCO_2_ has been of interest to researchers as some of the mechanisms by which they regulate stomatal dynamics seems to be the same. 

We have seen that eCO_2_ can mitigate drought-induced stress in plants through osmotic adjustment, changes in turgor pressure and changes in root shoot ratio, and the mechanism here is higher hydraulic conductance induced maintenance of higher relative water content (RWC). This is in addition to optimum water status being maintained by hydraulic conductance. On the other hand, when there is an interaction of eCO_2_ with drought, we are faced with the question as to what exactly is contributing to the stomatal dynamics. Is it the eCO_2_-induced changes in the stomata, or is it the drought-induced ABA production that is instrumental, or is it an action of both these agents in tandem?

The mechanism and the effect become complex when we see that both ABA and eCO_2_ induce stomatal closure: in the case of ABA it is reasoned that closure is to prevent excessive loss of water during stress, and in the case of eCO_2_ the reason for the induced closure is debated. The complexity further increases when we see that under water stress conditions in eCO_2_ there can be a combined action of both ABA and eCO_2_. Further, taking the complexity to the next level is the differential effects of eCO_2_ seen in C_3_ and C_4_ plants where the responses are distinct and conserved within the photosynthetic types [19].

Two Tomato genotypes, one of them being a mutant deficient in ABA, were tested for responses of hydraulic conductance at eCO_2_ by [23]; they found that a reduction in the transpiration rate and a concomitant increase in the water use efficiency (WUE) was seen in the wild type and not in the mutant, clearly indicating a role of ABA in this response. On the other hand, both in the mutant and the wild type, increased water use and osmotic adjustment was seen, showing us that plant water consumption which also includes water transpired is not entirely controlled by ABA. This also shows that osmotic adjustment as a response to water stress can have several other metabolic players and can occur even in the absence of ABA. It is possible that the protective role of ABA under stress is regulated by a higher concentration of CO_2_ and is manifested in higher WUE and reduced transpiration rate. It is generally thought that the eCO_2_-mediated closure of stomata and the opening of stomata are independent of the ABA pathway; on the other hand, some signalling components of the ABA pathway have been implicated to work in tandem, suggesting that some of the components of the regulatory mechanism are shared [24]. 

The response triggered by both these agents eCO_2_ and water deficit is interconnected, where ABA is shown to modulate and also regulate the effect of eCO_2_. Water deficit stress is known to have a stronger effect on stomatal conductance as compared to eCO_2_ and when in combination with water deficit stress causes a larger decrease in the stomatal conductance which could be an additive effect.

We see here that ROS is a necessary intermediate for ABA-mediated stomatal action in both eCO_2_ and water deficit, and while ROS is a well-known response under water stress. It is not so in eCO_2_, so the condition of ROS being a necessary intermediate for stomatal dynamics under sole eCO_2_ throws up some mechanistic challenges as to how this condition is satisfied, or if there is an alternative mechanism. This question, to an extent, justifies the certain degree of controversy that exists in the convergence of ABA and CO_2_ signalling [25]. 

SLAC1 is a membrane protein that is multispanning and is mainly expressed in the guard cells; it has an important role in the regulation of ion homeostasis in the cell and is also involved in S-type anion currents. It is a ubiquitous protein for effecting stomatal closure under various environmental signals like eCO_2_, water deficit stress, ozone, light regimes and many more. Studies have shown that SLAC1 activity loss due to mutation continues to affect CO_2_ responsiveness in stomatal closure and does not affect the same way under ABA, suggesting the presence of an ABA independent signalling network under eCO_2_ conditions to cause stomatal closure. This adds to the intrigue in the signalling response, and possible answers can be found when we can characterize the full complement of guard cell signalling sensors [26,27,28,29,30]. The role of guard cell chloroplasts in regulating CO_2_ has also been extensively studied; they are not directly involved in the control of stomatal closure as induced by CO_2_, as it is controlled by the conversion of CO_2_ to protons by carbonic anhydrases with HCO_3_ being the primary signalling molecules bringing about changes in the proton concentration, and as a result, controlling the opening and closure of the stomata [31,32,33,34]. 

We already know that the lower the concentration of CO_2,_ the more the opening of stomata, and as it goes higher the stomata start to close; CO_2_-induced closure is mediated by Ca^2+^ and protein phosphorylation, and the specific phosphorylation events are set into motion by signal transduction by Calcium-dependent protein kinases (CPKs) and calcineurin- B-like proteins (CBLs), with the secondary messenger being Ca^2+^ [35]. Ca^2 +^ also has an ABA modulated and accelerated response, hence Ca^2+^ transporters and proteins may have a twin function connected to both eCO_2_ and ABA [36,37,38]. Recent research has shown a role for both eCO_2_ and ABA in stomatal closure. 

**Figure 1:**
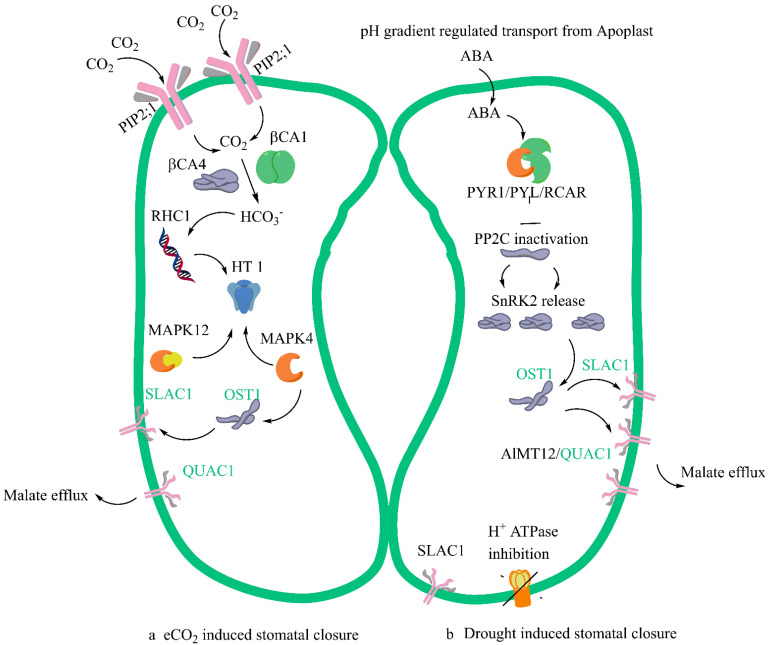
A simplified model of stomatal closure effected by eCO_2_ (**a**) and ABA (**b**) with commonality and convergence in the mechanism shown in green. Several Aquaporins felicitate the entry of CO_2_ in guard cells. Plasma membrane intrinsic protein (PIP2;1) aquaporin that facilitates water transport across the cell membrane and carbonic anhydrases (b CA4 and b CA1) interact leading to the in-creased formation of Bicarbonate (HCO^3−^). The multidrug and toxic compound extrusion (MATE)-type transporter RESISTANT TO HIGH CARBON DIOXIDE 1 (RHC1) gene product senses HCO_3_ signalling. Carbon Dioxide and Bicarbonate together act as signal transduction molecules. Under eCO_2_ the possible action would be the activation of MPK12 and MPK 4 resulting in the inhibition of expression of protein kinase HIGH LEAF TEMPERATURE1 (HT1). When ABA enters the guard cells, in the ABA-mediated closure PYRABACTIN RESISTANCE1 (PYR1)/PYR1-LIKE (PYL)/REGULATORY COMPONENTS OF ABA RECEPTORS (RCAR) it interacts with Type 2C protein phosphatases (PP2Cs) and inhibits them. The proton translocating ATPase is inhibited in the process by ABA and this prevents proton entry into the guard cells and regulates its pH. There is a subsequent release of Ca^2+^-independent protein kinases (SnRK2s). SLAC1 ion channel is phosphorylated by the activation of SnRK2 and calcium dependent protein kinases (CPK). The convergence step under both eCO_2_ and ABA is the activation of Rapid type ion channel aluminium-activated malate transporter 12/quickly activating anion channel 1 (ALMT12/QUAC1) which leads to the turgor dynamics and K^+^ ion efflux and resultant stomal closure.

The common pathway or overlap, or sometimes called the convergence point in the mechanism of stomatal closure, involves three different events. The first is the signal perception by SLAC1 of HCO_3_ where there is an involvement of several protein kinases, and this signalling activates the SLAC1 anion channel. The signalling is downstream of the Open Stomata 1 and Sucrose non-fermenting related Kinase (1OST1/SnRK1) pathway [15,39,40]. The mechanistic differences in the eCO_2_-mediated stomatal closure and ABA-mediated stomatal closure are shown in Figure 1.

### 2.3. Water Relations

In a study with field experiments and process-based simulations [41], the authors have shown that CO_2_ enrichment contributes to decreased water stress and also contributed to higher yields of maize under restricted water conditions. They showed from their studies that elevated CO_2_ decreases transpiration without any effect on soil moisture and at the same time it increases evaporation. Modelling has shown that water stress is reduced to an extent of 37 per cent under elevated CO_2_, a simulated increase in stomatal resistance being the reason for this.

Some of the effects of water stress in combination with elevated CO_2_ can be understood when we see the effects observed in Free Air CO_2_ enrichment (FACE) experiments. In maize elevated CO_2_ reduces transpiration and this, in turn, contributed to the increase in soil moisture and evaporation. In a simulated study [41] it was seen that transpiration was reduced by 22 per cent in the first year of the experiment. In another study [42] the authors showed that in a FACE experiment transpiration in maize was reduced significantly under 550 ppm CO_2_ concentration. Daily sap flow and vapour pressure deficit (VPD) of maize were investigated [43], and it was seen that whole-plant transpiration was reduced by 50 per cent in drought as compared to wet in ambient CO_2_ concentrations, and 37 per cent reduction was observed in elevated CO_2_ concentration of 550 ppm. Enrichment of CO_2_ did not affect sap flow under drought and a 20 per cent decrease was seen under wet conditions. Maize under elevated CO_2_ had a higher transpiration rate which was due to lower sap flow in the preceding period when plant-available soil water was minimum, this shows that reduction in canopy transpiration by elevated CO_2_ can delay the effects of water stress and can contribute to increased plant biomass production.

Another study [44] on the physiological response of two C_3_ and C_4_ mechanisms syndromes, examined Napier grass (*Pennisetum purpureum* Schumach × *Pennisetum glaucum* (L.) R. Br) and hydric common reed grass (*Phragmites australis* (Cav.) Trin. Ex Steud); under water stress and elevated CO_2_ it was seen that there was a general response of increase in photosynthesis, reduced leaf water potential, and increase in transpiration in both the grass species. A contrasting response was seen in the two types of grass to elevated CO_2_ and water stress; the difference in the species response was due to the stomatal characteristics as evidenced by the changes in transpiration rate and osmotic adjustment. Water status adjustment by modification of xylem anatomy and hyrodolyic properties is a mechanism found in many plants, and its relationship with the observed effect of elevated CO_2_ to increase plant water potential via reduced stomatal conductance and water loss has been studied [45]. One of the known adaptations to water stress by plants is to maintain high water potential and turgor pressure under water-deficient conditions. The authors saw in their study that water deficit significantly decreased xylem vessel diameter, conduit roundness and stem cross-section area, and it was seen that these impacts of water deficit were relieved at elevated CO_2_. In another study [46] where the adverse effects of the drought were studied on soyabean under elevated CO_2_, the authors found that elevated CO_2_ increased WUE contributing to countering drought, but they did not find any positive effects on osmotic adjustments.

The effects of Elevated CO_2_ individually and in combination with a water deficit in soyabean were studied [47]. In instantaneous water stress treatment, elevated CO_2_ reverted the expression of genes related to stress, transport and nutrient deficiency that was induced by water stress; the interaction of drought and elevated CO_2_ affected the expression of genes with physiological and transcriptomic analysis showing that elevated CO_2_ can mitigate the negative effects of water stress in soyabean roots.

## 3. Dry Matter Production

### 3.1. Photosynthesis and Growth

In addition to understanding the acclimation pattern of plants under a combination of water stress and elevated CO_2_, future yield prediction can also be done under the changing climate scenario from precise data on the effects of elevated CO_2_ and drought on biomass and soil water conditions. Growth modelling under these conditions has contributed to our knowledge of these effects. Under sufficient water supply, C_3_ crops recorded increased yield under elevated CO_2,_ whereas C_4_ crops did not show much change in the yield. A 10–15 percent increase in biomass has been seen in C_3_ crops under FACE experiments due to the CO_2_ fertilizing effect [48,49]; on the other hand, C_4_ crops maize and sorghum did not respond similarly under water sufficient conditions [50,51]. In a study [52], it was seen that adverse effects of heat and drought were alleviated by improved water relations under elevated CO_2_. The authors also saw that the mechanism of photosynthesis reduction under the combination of heat and drought was due to increased drying of soil and a decrease in stomatal conductance.

**Figure 2:**
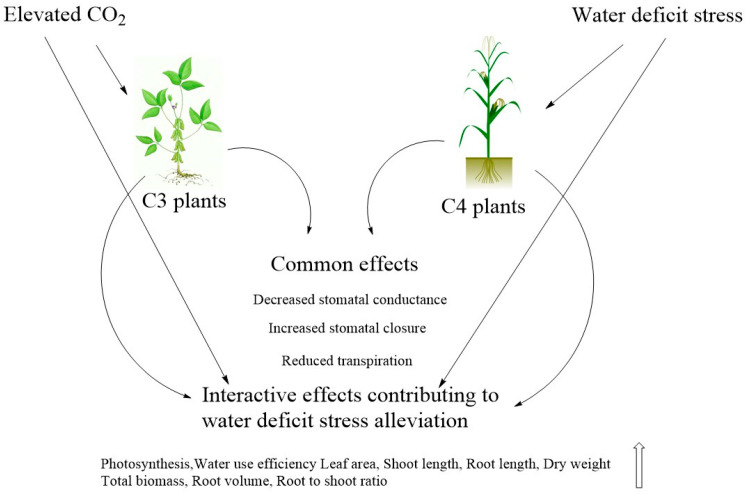
Schematic representation of individual and interactive effects of water deficit stress and eCO_2_ in C3 and C4 plants.

In C_3_ plants, the most prominent effect is the increased photosynthesis due to the higher availability of CO_2_ to rubisco and due to reduction in photorespiration [53,54]. The effects of eCO_2_ on C_3_ plants have been widely studied, and from a recent comprehensive meta-analysis [55] it is seen that under eCO_2_ that leaf biomass per unit leaf area (LMA) increases with a slight decrease in leaf N content. The effect on photosynthesis is mainly due to higher concentrations of CO_2_ in the vicinity of Rubisco and thus an increased affinity and reduced photorespiration. Some of the other effects were an almost doubling of photosynthetic rate at higher concentrations of CO_2_ (1000 ppm range), with a halving of stomatal conductance and transpiration per unit lead area [55]; this was seen when the stomatal density as such was not affected, implying that the reduced transpiration is due to closure of stomata (the mechanism of which is discussed in another section in this review). This increase in photosynthesis accompanied by reduced transpiration leads to higher WUE, which is also a characteristic of C_4_ plants under eCO_2_ and it is one of the reasons for eCO_2_ under water deficit conditions being less detrimental to the plant as against water-deficit stress in isolation. A schematic representation of the interconnected effects of both eCO_2_ and water deficit stress in isolation and in combination in both C3 and C4 crops is given in Figure 2.

In a study on Macauba palm [56], the authors investigated the effects of elevated CO_2_ and drought on photosynthesis, and found that at elevated CO_2_ the plants could recover more from water stress due to increased Rubisco carboxylation rate and electron transport rate, thus preventing reduction in total dry matter production. The authors noted that drought and increased CO_2_ affected stem length and total dry matter production, and it was seen that at elevated CO_2_ there was no reduction in stem length and total biomass due to drought.

In coffee, it was seen by authors [57] that at 723 ± 83 ppm concentration of CO_2_ for a period of seven months there was increased biomass accumulation even under water deficit treatments with reduced rates of photorespiration and oxidative pressure under drought. The plants under drought and elevated CO_2_ showed high respiratory carbon flux, which is high respiration rates and an energy status that supported increased root growth under drought. These results show a new mitigating method of elevated CO_2_ for the maintenance of photosynthetic performance under drought. Other studies have shown that in soyabean [58] drought effect on photosynthesis was not alleviated by elevated CO_2_, the authors found that net photosynthetic rate and chlorophyll b content reduced under drought and elevated CO_2_. Another study [48] evaluated biomass accumulation in long-term experiments under elevated CO_2_ and drought, and the authors reported that there was a multiple response pattern and suggested long-term experiments to access the future impact of climate change. One of the ways eCO_2_ increases the biomass is by increasing Leaf Area Index (LAI) at early developmental stages leading to increased utilization of incident radiation and in turn higher carbon fixation [59,60].

Among the growth parameters, relative growth rate (RGR), net assimilation rate (NAR), and leaf area ratio (LAR) are the key parameters that have shown consistent increase due to eCO_2_. Under water deficit, we see that all these parameters are negatively affected, and the positive effect of CO_2_ is shown to protect the plants from adverse effects of drought in isolation. Under eCO_2,_ the higher NAR is compensated by reduced LAR which in turn is caused by the decrease in specific leaf area (SLA); this has been consistent with all the plant parts above ground and below ground [55,61,62].

The observed negative response or lack of acclimation of photosynthetic carbon fixation to long-term eCO_2_ can be due to enrichment-induced disruption of RuBP and Pi regeneration in the leaves, which can alter photosynthetic rates effectively lowering it down. This long-term effect has not been studied well for reasons mainly that most experiments with eCO_2_ are for two crop seasons for 3–5 years which does not simulate the environmentally relevant concentration of CO_2_ over a long term, which constitutes the future scenario of climate change.

### 3.2. Malate Maelstrom

Some of the major difference in the response to eCO_2_ and drought among plants are due to the operation of different types of photosynthetic pathways in plants, namely the C_3_ and C_4_ pathways. The C_4_ plants are adapted to low concentrations of CO_2_ in the atmosphere as they have a special anatomy called Kranz anatomy, which helps them concentrate CO_2_ within the cells and reduce the oxygenase activity of RuBPcase thus reducing photorespiration and increasing photosynthesis. Consequently, the C_4_ plants are not benefitting from increased CO_2_ concentrations in the atmosphere as are C_3_ plants. On the other hand, the temperature optimum for C_4_ plants is high, and so under eCO_2_ and drought conditions this adaptability confers a degree of tolerance to these plants. The main enzymes of the C_4_ plant that play an important role in photosynthesis are the decarboxylating enzymes, NAD-dependent malic enzyme (NAD-ME), and NADP-dependent malic enzyme (NADP-ME). They are of diverse phylogenetic origin, and they are present in mitochondrial isoforms in C_3_ plants not playing a role in carbon fixation as they do not have the oxaloacetate decarboxylase (OAD) action [63,64,65,66]. In higher vascular plants the Malic enzymes are seen as a widely distributed isoforms in the plastids and mitochondria; they have multivarious functions ranging from deference, conferring tolerance to abiotic stresses, metabolic control, and stomatal dynamics [65,66,67,68].

**Figure 3:**
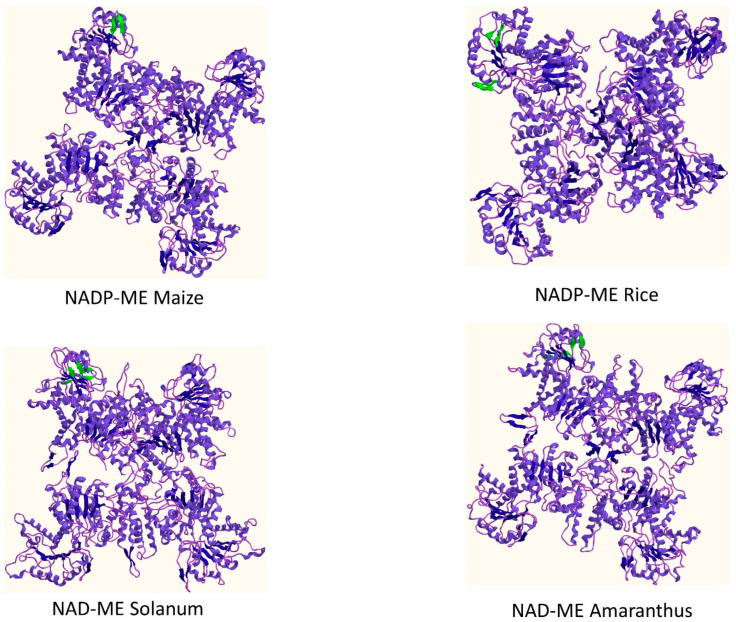
Homology models of NADP-ME and NAD-ME enzymes with their beta hairpin bends shown in green.

In the NAD-ME type plants, the enzyme has the main function of decarboxylation of malate to produce pyruvate, NADH and CO_2_; this enzyme uses NAD^+^, does not decarboxylate Oxalo Acetic Acid (OAA) and is present in mitochondria. Drought and C_4_ enzymes, specifically the NAD and NADP malic enzyme action dynamics, have been well studied [14,67,69,70]. The possible mechanism can be that the entry of CO_2_ is restricted under eCO_2_ conditions due to reduction in stomatal density and closure, either due to eCO_2_ or drought; the ME in C_4_ can contribute to higher accumulation of CO_2_ in the bundle sheath cells, thus increasing photosynthesis, and in contrast to the commonly accepted view that C_4_ plants do not have a distinct advantage under eCO_2_ conditions. There is also evidence that ME has an important role in balancing out malic acid and decreasing the Reactive Oxygen Species (ROS) generation, thereby offering protection against oxidative damage [71]. Considering all these aspects of Malic Enzymes, we did a sequence search, alignment, and homology modelling of four different kinds of MEs to understand if there are possible structural differences in these different enzymes that can explain the functional diversity. It was seen that there were differences in the protein secondary structure (Table 1), and distinct hairpin bend differences in the structure of the enzyme (Figure 3).

The difference in the beta-hairpin motifs across these NAD-ME and NADP-ME can play an important role in the versatility of the structure and the enzyme action. Positional differences in the amino acids in the hairpins can explain substrate binding and affinity differences in these enzymes. Engineering these enzymes by CRISPR CAS genome editing techniques can be a way to change the activities of these enzymes in both C_3_ and C_4_ plants, and it can play an important role under both eCO_2_ and water deficit conditions.

### 3.3. Nutrients in a Nutshell

Nutrition limitation under eCO_2_ and water deficit conditions is one of the main reasons for lower dry matter production apart from the other stress-related responses. Adequate nutrient supply and an adaptive mode of transport into the plant system can alleviate these stresses in a big way. The long-term responses and short-term responses to both eCO_2_ and water deficit could be very different and even opposite in their manifestation. Critical inquiry into the mechanistic process involved here will throw light on possible ways by which these stresses can be tackled. In a study on the possible adaptive response of semi-dwarf durum wheat cultivars by physiological and molecular mechanisms [72], it was seen that elevated CO_2_ and water stress increased d15N, which was cultivar dependent, and the effect diminished as water stress increased. Shifts in N metabolism could reflect in decreased root-to-shoot translocation of N. The authors observed d13C increased under moderate stress irrespective of the CO_2_ concentration indicative of higher water-use efficiency. Phosphoenol Pyruvate Carboxylase (PEPC) expression was increased under water stress and elevated CO_2_ combination. Carbohydrates, which are the substrates for PEPC, increased under these stresses and this showed the role of PEPC in providing carbon skeleton for amino acid and lipid biosynthesis. It is seen that there is a transcript level coordination in C and N metabolism under a combination of water stress and elevated CO_2_. The dehydrin genes DHN11 and DHN16 showed changes in expression under water stress and elevated CO_2_ with genotype-dependent change in transcript levels, this shows that the interactive effects of both elevated CO_2_ and water stress varies according to the genotype in wheat.

In systems where there is no limitation for nutrients, and where there is a sufficient amount of nutrients either in the natural ecosystem or managed system, some species do not show any adaptation or acclimation to higher levels of CO_2_ [73]. There has been ongoing research on the reasons for this lack of acclimation to elevated CO_2,_ especially on an ecosystem scale, and the possible reasons are not clearly explained yet; although some explanations have been put forth among which is feedback inhibition in the source-sink relationship caused by increased carbohydrate loading in the leaves which can reduce photosynthesis [74].

The observed decrease in nitrate concentration in plants under eCO_2_ especially in the leaves can be explained by the lower rates of transpiration also observed at high CO_2,_ which causes a reduced soil solute mass flow along the plant’s above-ground parts starting from the roots in the soil plant atmosphere continuum [75,76]. The reduction could also be due to a down-regulation of nitrogen assimilation, differentially regulating N transporters which maybe dose-dependent [77,78].

A summary of effects of e CO2 and drought in plants is given in Table 2. A proposed mechanistic explanation for this is also reduced photorespiration which can reduce N assimilation [79], where the authors show that chloroplast vesiculation (cv) gene expression which destabilizes photosynthesis when silenced maintained the N assimilation status in plants under eCO_2_. The key point to note here is that the total N in the plant is increased under eCO_2_ because of the biomass increase seen in these conditions; this mechanism as such may not be a part of the mitigating factor when eCO_2_ and water deficit are in combination, as the reduction in the biomass induced by water stress is not fully compensated by eCO_2_ although the combination is seen to reduce the reduction caused by water deficit stress.

Adequate Phosphorus nutrition is an ameliorative agent under water deficit stress in eCO_2_ conditions. The mechanism by which P acts under eCO_2_ conditions is by increasing the concentration of soluble sugars and Pi and maintaining relative water content in the leaves. In a study [80] in field peas, the authors found that the stress tolerance index (STI) was higher in plants with adequate P under eCO_2_ conditions under water deficit, which exemplifies the interactive role of P and eCO_2_ as drought ameliorative combination. The role of P is to stabilize water relations, stomatal conductance, and transpiration rate synergistically with eCO_2_. It is instrumental in decreasing stomatal conductance and concomitantly reduces water loss through transpiration, thus increasing the transpiration efficiency under drought stress conditions. Total soluble sugars increase in leaves due to accumulation of leaf Pi under eCO_2_ and offers better disposition of plants to stave off drought-related stress than when in eCO_2_. The positive effects of Pi namely maintenance of high metabolic readiness to tackle low water status are enhanced under eCO_2_. A similar trend was seen in Potassium (K) supplementation under water deficit conditions [81]. The authors found that K deficiency impeded the ameliorative effect of eCO_2_ on drought. Nutrient sufficiency is an important prerequisite to realise the beneficial effects of eCO_2_ and the drought mitigatory effect of eCO_2_ is dependent on nutrient sufficiency.

**Table 2:** Summary of effects of Elevated CO_2_ and drought in plants.

Plant/Crop/Tree	CO_2_ Concentration	Water Stress Imposition	Effect	Reference
Poplar (*Populus* spp.)	700 ± 50 µmol mol^−1^	Soil drying cycle by withholding water	Reduced Gas exchange, decreased leaf conductance, increased photosynthesis, increased transpiration efficiency	[12]
Wheat (*Triticum aestivum*)	400 μmol mol^−1^, 790 μmol mol^−1^	Progressive restriction of water from 10 percent to 60 percent pot capacity	Reduced plant biomass, Stomatal conductance and carbon isotope signature indicated water saving strategy. PEPC expression increased	[72]
*Tabernaemontana divaricata*	1000 μmol mol^−1^, 700 μmol mol^−1^	70 percent of field capacity (FC) for 4 days and 30 percent of FC for 4 days	Increase in stomatal conductance (g_s_), plant height (PH) and plant girth (PG)	[82]
Maize (*Zea mays*)	550 μmol mol^−1^	Half water in water stress treatment compared to control	37 percent reduction in whole plant transpiration	[43]
Napier grass (*Pennisetum purpureum* Schumach × *Pennisetum glaucum* (L.) R. Br) and hydric common reed grass (*Phragmites australis* (Cav.) Trin. Ex Steud)	563 ± 6.7 μmol mol^−1^ 541 ± 6.9 μmol mol^−1^ 601 ± 9.1 μmol mol^−1^	Withdrawing irrigation	Increase in Photosynthesis, reduced leaf water potential and increase in transpiration	[44]
Maize (*Zea mays*)	700 μmol mol^−1^, 900 μmol mol^−1^, and 1200 μmol mol^−1^	deficit irrigation	Decreases in stomatal conductance and reduced transpiration rate	[45]
Soyabean (*Glycine max*)	Ambient + 200 μmol mol^−1^	35–45 percent of RWC	Elevated CO_2_ enhanced the resistance to drought by improving the capacity of photosynthesis and WUE in soybean leaves	[46]
*Pinus halepensis* (Aleppo pine)	867 ± 157 μmol mol^−1^	10 Percent Relative Substrate Water Content	Under drought, the effect of CO_2_ on WUE was pronounced, with intercellular CO_2_ being increased near stomatal closure	[83]
Lemon (*Citrus limon*)	650 and 850 μmol mol^−1^	leaf water potential of −3.5 MPa	Stomatal downregulation at elevated CO_2_ reduced water-use but not photosynthesis.	[84]
Soybean (*Glycine max*)	800 μmol mol^−1^	water deficit was applied by randomly moving plants out of the hydroponic solution exposing the roots to ambient- or elevated-air	Responses of soybean roots to short-term water deficit are buffered by Elevated CO_2_	[47]
Cassava (*Manihot esculenta* Crantz)	750 μmol mol^−1^	Stopping irrigation for 7 days	Elevated CO_2_ reduced the negative effect of drought on transpiration, water use efficiency, all growth measures and harvest index.	[85]
Faba bean (*Vicia faba* L.)	550 μmol mol^−1^	Water was withheld until 30 percent FC	Elevated CO_2_-induced stimulation of nodulation and nodule density helped maintain N_2_ fixation under drought	[86]
Andiroba (*Carapa surinamensis*)	700 μmol mol^−1^	50 percent field capacity	Whole-plant water-use efficiency (WUE) improved under combination treatments	[87]
*Hymenaea stigonocarpa* Mart. ex Hayne, *Solanum lycocarpum* A. St.-Hil. and *Tabebuia aurea* (Silva Manso) Benth. and Hook. f. ex S. Moore	700 μmol mol^−1^	Water stress was introduced three times during the experiment by halting irrigation 1 month before the fourth (360 days old), fifth (450 days old) and sixth (540 days old) morphophysiological surveys	Water stress decreased biomass production under high CO_2_	[88]
Grapevines (*Vitis labrusca*)	800 μmol mol^−1^	Stopping irrigation	Elevated CO_2_ delayed drought effects on both net photosynthetic rate and Rubisco activity for four days, by reducing stomatal conductance, transpiration, and stomatal density	[89]
*Brassica napus*	800 μmol mol^−1^	Withholding water for 7 days	Elevated CO_2_ diminished the adverse effect by improved water relations	[52]
Maize (*Zea mays*)	550, 700, and 900 μmol mol^−1^	Deficit irrigation	Photosynthetic rate in elevated CO_2_ concentrations was higher under Deficit irrigation than under regular irrigation.	[90]
*Acrocomia aculeata*	700 μmol mol^−1^	Water withholding	Higher Rubisco carboxylation rate (V_c_ max) and electron transport rate (J max) contributed to recovery from drought	[56]
Cucumber (*Cucumis sativus* L.)	800 ± 20 μmol mol^−1^	ψw = −0.05 MPa and ψw = −0.15 with PEG 6000	Higher photosynthetic performance and increased grana thickness under moderate drought stress, increased palisade cells length and chloroplasts number per palisade cell under severe drought stress.	[91]

## 4. Future Perspectives

Deficit irrigation to economize water use and to induce acclimation by plant physiological adjustments is an approach that can be advocated to counter the adverse effects of changing climate; our mini-review here shows that this can be an important strategy in future agriculture under elevated CO_2,_ which effectively decreases the impact of low soil water on photosynthesis and in turn biomass accumulation and yield in crops. Plant water relations are mainly affected by gas exchange and stomatal physiology which in turn is affected by elevated CO_2_ and drought and there is a complex manifestation when these stresses act in combination; these are the critical factors when the goal is to evolve climate ready cultivars. To devise strategies for adaptation in crops in agricultural systems, we have to understand and elucidate how these processes operate across a range from ecosystems to organismal level and from cellular and biochemical to the molecular level. Adaptation in agriculture to changing climate is occurring all over the world; the practices should now be based on the findings that eCO_2_ under drought and water stress conditions can be effective in alleviating the effects of climate change. There is a consensus and better understanding of effects now that can be put to use for tackling climate-related effects on crop production.

One of the important facets that have come out of this mini-review is that most of the effects observed need to be looked into with a mechanistic perspective, to arrive at correct inferences that can help us move ahead with the goal of evolving climate-ready cultivars. In many of the studies, the casual association is observed that needs further investigation; we trust that this mini-review will invigorate researchers.

## Figures and Tables

**Table 1:** Secondary structure difference between selected representative NADP-ME and NAD-ME in different plants.

Enzyme	Sheets	Beta Alpha Beta Units	Beta Hairpins	Beta Bulge	Strands	Helices	Helix-Helix Interaction	Beta Turns	Gamma Turns
NADP-ME Maize	4	5	1	0	14	33	47	36	3
NADP-ME Rice	4	5	2	0	15	33	44	33	4
NAD-ME Solanum	3	5	2	1	14	35	44	47	6
NAD-ME Amaranthus	4	5	2	1	14	36	45	39	5

## Data Availability

The original contributions presented in the study are included in the article, further inquiries can be directed to the corresponding author.
